# Three-Body Abrasive Wear Behavior of WC-10Cr_3_C_2_-12Ni Coating for Ball Mill Liner Application

**DOI:** 10.3390/ma15134569

**Published:** 2022-06-29

**Authors:** Qiang Hu, Dehui Ji, Mingxue Shen, Hui Zhuang, Hailong Yao, Huoping Zhao, Hui Guo, Youliang Zhang

**Affiliations:** 1 Institute of Applied Physics, Jiangxi Academy of Sciences, Nanchang 330095, China; huqiang1225@163.com (Q.H.); zhuanghui1120@163.com (H.Z.); guohui@jxas.ac.cn (H.G.); zhangyl0923@163.com (Y.Z.); 2 College of Materials Science & Engineering, East China Jiaotong University, Nanchang 330013, China; jidehui_1990@163.com (D.J.); zhaohphust@126.com (H.Z.); 3 Jiangxi Province Engineering Research Center of Materials Surface Enhancing & Remanufacturing, School of Mechanical and Materials Engineering, Jiujiang University, Jiujiang 332005, China

**Keywords:** HVOF coating, particle size, three-body abrasive wear, wear mechanism

## Abstract

Carbide coatings are frequently used to improve the wear resistance of industrial components in various wear environments. In this research, aiming at the service characteristics of easy wear and short service life of ball mill liners, WC–10Cr_3_C_2_–12Ni coatings were prepared by supersonic flame spraying technology (HVOF). The reciprocating sliding tests were conducted under four different WC particle size conditions, and the differences in the tribological behavior of the coatings and three–body abrasive wear mechanism were obtained. The findings reveal that the average nanohardness of the WC–Cr_3_C_2_–Ni coating is nearly five times greater than that of the steel substance. The COF of tribo-pairs decreases and then increases as the particle size increases. In the case of no particles, the surface of the coating is slightly worn, with fatigue and oxidative wear being the primary wear mechanisms. Small particles (1.5 μm and 4 μm) are crushed and coated on the coating surface, in which the extremely fine particles are plasticized to form friction layers that have a protective effect on the coatings. The protective effect of the particles disappears as the particle size increases and is replaced by a powerful chiseling effect on the coatings, resulting in serious material loss. The particle size has a direct relationship with coating wear.

## 1. Introduction

As common grinding equipment, the ball mill is mainly composed of a cylinder, grinding ball, liner, and feeding device, and is widely used in metallurgy, ceramics, construction, chemical and pharmaceutical industries [1,2,3]. As a key component to protect the cylinder and enhance the crushing efficiency of materials, ball mill liners will be directly impacted and worn by grinding balls and materials during the working process, resulting in a huge consumption. Therefore, improving the wear resistance of ball mill liners and prolonging their service life has become one of the most urgent problems to be solved in the current engineering field. With the rapid development of material grinding technology, higher requirements are placed on the wear resistance of lining materials. At present, the development of coating materials with excellent wear resistance on the liner has become a research hotspot [4,5].

Proper material selection is essential to enhance performance and withstand harsh operating conditions. The coatings obtained by the application of supersonic flame spraying (HVOF) technology have the advantages of high density and good bonding strength, and are widely used in spraying cemented carbide coatings on parts for surface strengthening treatment [6,7]. Among them, WC–based coatings, such as WC–Co, WC–Ni and WC–CoCr, have high-fatigue resistance, oxidation resistance at high temperatures, and corrosion resistance [8,9]. Nevertheless, in the WC–Ni alloy, the solubility of W in Ni is more than 17%, and the performance of the alloy is very brittle, which once limited its application range [10]. Therefore, abundant research work has been carried out to improve the brittleness, and found that Cr_3_C_2_ is not easily able to produce brittle phases during deposition, and is well combined with steel substrate materials. Thus, the coating has good mechanical toughness. In addition, Cr_3_C_2_ can prevent the WC granules from growing during the sintering process, improving the overall hardness [11,12,13]. Even at a temperature above 650 °C, the obtained WC–Cr_3_C_2_–Ni coatings have excellent anti-oxidation and wear resistance [14,15]. On the other hand, among the powder components available on the market, Cr_3_C_2_ is more readily available and cheaper than Mo, Cr, Y and other components that can improve the overall performance of the coating, thus, the WC–Cr_3_C_2_–Ni coating has become the correct choice for many industrial applications.

However, in addition to the composition of the hard phases affecting the wear resistance of coatings, the contact state between the coatings and count-pairs, and the service environment are also important influencing factors [16,17]. In recent years, the three-body abrasive wear of the WC–based coatings has received extensive attention, and related research mainly focuses on the effects of the hard phase content, abrasive particle hardness and abrasive particle content on the tribological behavior of coatings [12,18,19,20]. In the three-body abrasive wear of the WC–20Cr_3_C_2_–7Ni coating, Wang et al. [19] discovered that the abrasive particle hardness is an essential factor that affects the wear mechanism, and that high-hardness particles diminish the wear resistance. Additionally, the wear resistance of the WC–Cr_3_C_2_–Ni coatings reduced as the sand content increased [20]. The extrusion and exfoliation of binder phases, as well as the fragmentation and exfoliation of hard phases, are the primary failure mechanisms of these coatings. Studies have shown that the deposition of abrasive particles in the pores of coatings alters the mechanical properties of the materials, such as hardness, shear modulus, elastic modulus, etc. The contact state between the tribo-pairs is different due to the participation of particles, which seriously affects the wear resistance of coatings [21,22,23]. Ghabchi et al. [24] found that more cracks and spalling develop on the worn surface when the abrasive particles are bigger than the pores of the WC–10Co4Cr coatings, resulting in a serious coating loss. Therefore, the tribological behavior of the coatings is significantly disturbed by the abrasive particles and has a distinct damage mechanism compared to dry friction. However, little research has been done on the impact of the particle size on the friction and wear properties of WC–Cr_3_C_2_–Ni coatings, and the wear mechanism remains unclear.

Therefore, the WC–10Cr_3_C_2_–12Ni coating was prepared using the HVOF spraying technology in order to improve the wear resistance of ball mill liners. Considering that ball mill liners serve in the three-body abrasive wear condition, the effects of particle sizes on the tribological behaviors and wear mechanism of the WC–Cr_3_C_2_–Ni coating was investigated, so as to provide theoretical guidance for the selection of a wear-resistant coating and the life extension of ball mill liners.

## 2. Materials and Methods

### 2.1. Coating Preparation

The raw materials for preparing the coating are WC powder (0.8–2.5 μm), Cr_3_C_2_ powder (0.6–1.5 μm) and Ni powder (1–3 μm). After ball milling and mixing for 10 h, the WC–10Cr_3_C_2_–12Ni–mixed powder was prepared by spray drying with a Cr_3_C_2_ content of 10%, Ni content of 12%, and the rest of the WC powder. Then, the mixed powder was sintered at 1140 °C for 2 h, and the ideal particle size distribution was obtained by sieving. The sintered powder was coated with the high-speed flame spraying equipment (HVOF, CH–2000, Xi’an Jiaotong University, Xi’an, China) on the substrate material-20# carbon steel, and the WC–10Cr_3_C_2_–12Ni coating samples were obtained. Before spraying, the surface of the substrate materials must be de-greased, with the rust removed, sandblasted, and cleaned. The process parameters of the HVOF are shown in Table 1. The surface roughness of the prepared coating samples was polished to 0.2 μm and cut into small pieces of 15 mm × 20 mm × 6 mm for the friction and wear test. Before and after the test, the samples were ultrasonically cleaned with absolute ethanol and air-dried.

### 2.2. Test Method

**Figure 1:**
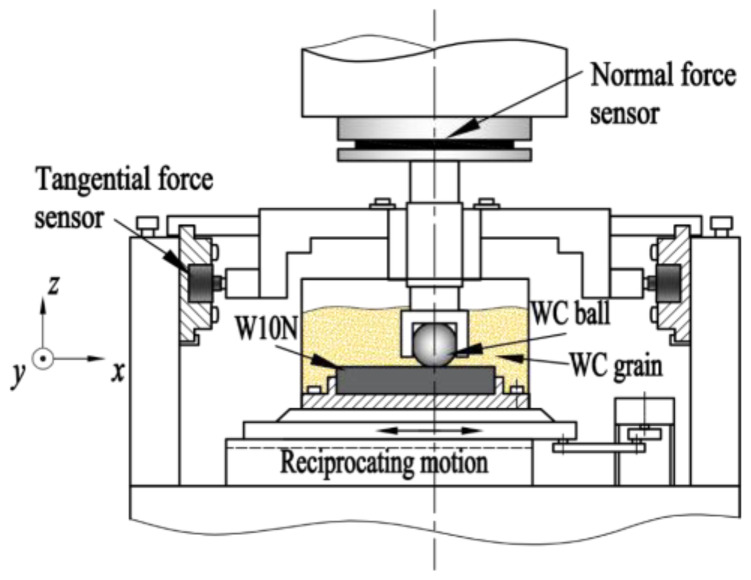
Schematic diagram of the friction test device.

**Figure 2:**
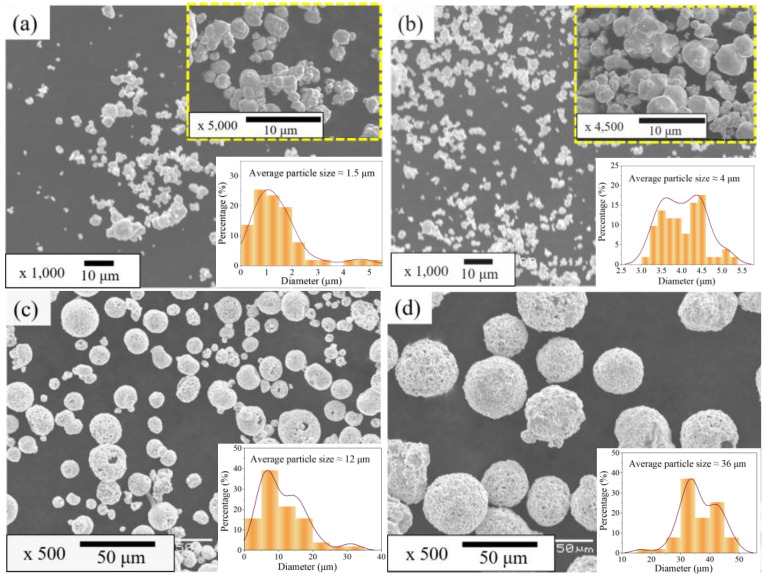
SEM images of WC particles of different sizes: WC particles with an average particle size of (**a**) 1.5 μm; (**b**) 4 μm; (**c**) 12 μm; (**d**) 36 μm.

The reciprocating sliding friction and wear performance of the coatings were simulated using the UMT-3 multifunctional friction and wear tester-modified abrasion test rig (based on ASTM G133) under various abrasive particle size conditions. Figure 1 shows the schematic diagram of the friction test device. The upper sample is a WC ball with a diameter of 7.968 mm and a nanohardness of 22.9 GPa, which is clamped and fixed by the upper clamp, and the lower sample is the WC–Cr_3_C_2_–Ni coating, which is installed in the lower clamp device and is driven by the drive system for a continuous reciprocating motion. Four kinds of WC particles, with an average particle size of 1.5 μm, 4.0 μm, 12.0 μm, and 36.0 μm, were used as the third-body abrasive particles, and the particle-free condition was used as the control group. The morphology of four WC particles is shown in Figure 2. During the experiment, the particles filled the entire tribo-pair space. Additionally, the test parameters are presented in Table 2. All tests were conducted at room temperature (23 ± 1 °C) with a relative humidity of 55 ± 2%. To assure the reproducibility of the experimental results, all abrasive wear experiments were repeated three times under the same conditions. During the test, the data acquisition system could collect data, such as the tangential force, normal force, frequency and time in real-time, and obtained the friction coefficient through calculations.

### 2.3. Analysis and Testing

An optical microscope (OM, OLYMPUS, BX53, Tokyo, Japan) was used to observe the cross-section and surface microstructure of the WC–Cr_3_C_2_–Ni coatings, and the hardness change in the coating section was measured by a nanoindentation tester (NHT3, Anton-Pear, Graz, Austria) with a maximum contact load of 50 mN and a holding time of 10 s. A three-dimensional profiler (Zygo, ZeGageTM Pro HR, Middlefield, CT, USA) was applied to measure the two-dimensional profile and three-dimensional topography of the coatings and the three-dimensional topography of the counter WC balls. The wear volume of the coatings is derived from the averaged 10 values of the linear integral of length over the coating two-dimensional contour area. A scanning electron microscope (SEM, SU8010, Hitachi, Tokyo, Japan) was used to observe the microstructure and damaged morphology of the coating wear scars, and the element distribution on the wear scar surface was evaluated using an energy dispersive spectrometer (EDS, Xflash6160, Bruke, Berlin, Germany). The phase composition of the wear scar micro-domains (0.5 mm × 0.5 mm) of the coatings was analyzed by an X-ray diffractometer (Rigaku, Rigaku Rapid IIR, Tokyo, Japan).

## 3. Results

### 3.1. Coating Microstructure and Hardness

**Figure 3:**
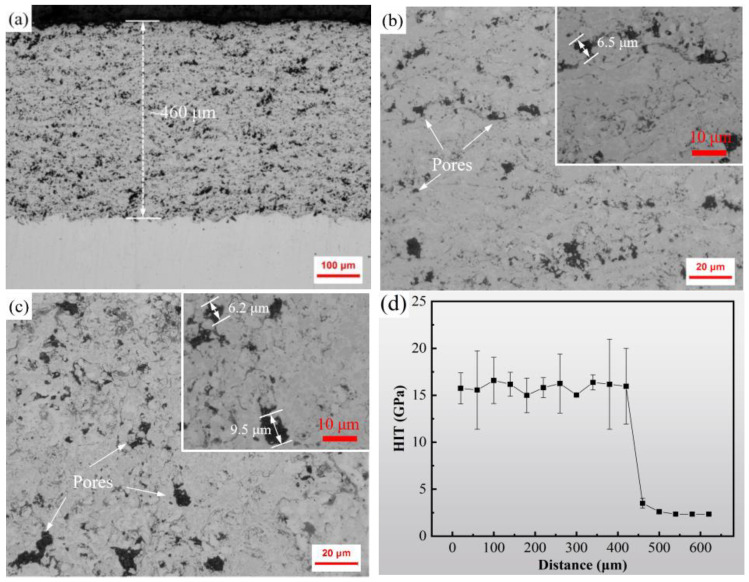
Microstructure and nanohardness of HVOF sprayed WC–Cr_3_C_2_–Ni coating: (**a**,**b**) the cross-section morphology; (**c**) surface topography; (**d**) nanohardness of cross-section.

The microstructure and nanohardness of the WC–Cr_3_C_2_–Ni coating obtained by the HVOF spraying are shown in Figure 3. The coating, with a thickness of about 460 μm, is tightly bound to the substrate (see Figure 3a), and the particles are uniformly distributed. It can be seen from Figure 3b,c that there are no microcracks except for the pores on the coating cross-section and surface, suggesting that the HVOF powder melted uniformly and the binder and hard phases combined well. Additionally, the pore size is mostly larger than the small particle size. However, the pore structure is one of the common characteristics of thermal spraying coatings. On the one hand, some sprayed droplets are prone to generating holes when they are sprayed onto surface materials or bounced, or the droplets themselves carry holes to cause pore solidification during deposition [25]. On the other hand, the decarburization reaction during the coating deposition process will also generate pores [26]. Figure 3d presents the variation curve of the nanoindentation hardness with a depth in the cross-sectional direction of the coating. The average nanohardness of the coating is about 16 GPa, which is nearly five times that of the 20# steel substrate.

### 3.2. Friction Coefficient

**Figure 4:**
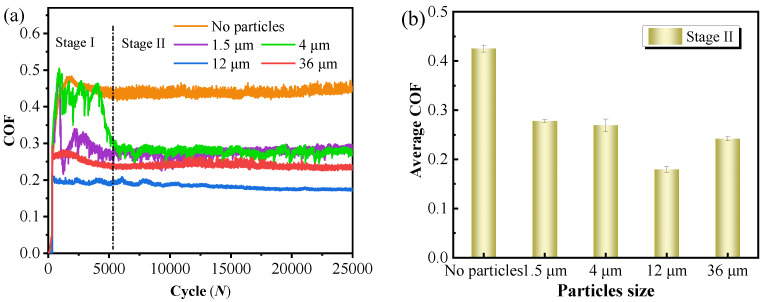
COF under different particle size conditions: (**a**) time-varying curve of COF; (**b**) the average COF of stage II.

The time-varying friction coefficient (COF) of the tribo-pairs and the average friction coefficient (A-COF) in the stable stage under the different particle size conditions are displayed in Figure 4. It was discovered that as the cycle numbers increase, the COF generally exhibits the characteristic of a sharp increase at first, followed by a slow decrease, and reaches a plateau after about 5100 cycles (Figure 4a). For the convenience of explanation, the COF is mainly divided into two stages: the running-in transition stage (I) and the stabilization stage (II).

In stage I, the COF fluctuates greatly under the conditions of 1.5 and 4 μm particles, especially for the 4 μm particles, while the fluctuation is smaller under the conditions of 12 μm and 36 μm particles. For illustration purposes, particles of 1.5 μm and 4 μm are defined as small-sized particles, while particles of 12 μm and 36 μm are defined as large-sized particles. The small-sized WC particles have agglomeration and poor fluidity (Figure 2a,b), making them difficult to enter into the tribo-interface. In the early stage of friction, direct contact between the tribo-pairs results in a higher COF. Compared with the 4 μm particles, the 1.5 μm particles invaded the tribo-interface earlier, hence, the COF reached a stable level in a shorter time. In the initial stage of friction, only a small number of 4 μm particles enter the interface, resulting in the COF being consistent with the particle-free condition. However, it is unavoidable that a small number of particles can still enter the tribo-interface, hence, the COF fluctuates more violently than that under the no-particle condition. Furthermore, with the increase in cycle numbers, the abrasive enters until the friction interface reaches a stable state, and the COF is reduced to a certain extent and remains stable. For the two kinds of large-sized particles, the COF is relatively stable in stage I without huge fluctuations. This is attributed to the fact that the large-sized particles without agglomeration are easy to enter and fill in the tribo-interface to reach a stable contact state at the initial cycle stage.

In stage II, the A-COF between the tribo-pairs generally decreased at first and then increased with the increasing size of particles involved in the wear. The A-COF in the particle-free condition is the highest, at about 0.43, while two small-sized particles decreased by about 35% and 37%, respectively. The A-COF reaches a minimum at the particle size of 12 μm. This may be attributed to the aggregation of abrasive particles after entering the tribo-interface, which isolates the direct contact between tribo-pairs, and a rolling friction condition is then established in the local region of the contact surface. Furthermore, as the particles of 12 μm are more likely to form a rolling state at the interface, the shear force is smaller and A-COF is reduced. However, a larger force is required if the abrasive particles of 36 μm are to be pushed to move on the interface, hence, the A-COF tends to increase.

### 3.3. Surface Morphology of Coatings

**Figure 5:**
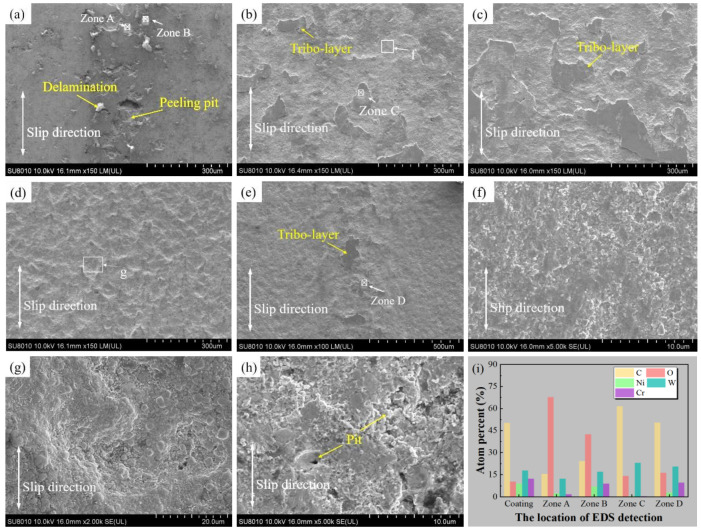
Surface morphology and EDS analysis of coating wear scars under different sizes of particle conditions: (**a**) no particles; (**b**) 1.5 μm; (**c**) 4 μm; (**d**) 12 μm; (**e**) 36 μm; (**f**) the enlargement of the region in (**b**); (**g**) the enlargement of the region in (**d**); (**h**) 36 μm, local area without tribo-layers; (**i**) the location of EDS detection.

The surface morphology and element composition of the coating wear scars under various particle size conditions are shown in Figure 5. Under the particle-free condition, a high number of spalling pits and peeling layers appeared on the coating surface (Figure 5a). The elemental compositions of Zone A and Zone B were analyzed by EDS, and it was found that the peeling layer was mainly composed of oxides (Figure 5i), indicating the existence of oxidation wear. Under the condition of small-sized particles, the worn surface is mostly covered by fine particles (Figure 5f) and tight and smooth tribo-layers, as shown in Figure 5b,c. According to the EDS analysis, the tribo-layers and other areas are primarily composed of W and C elements (Zone C in Figure 5i), indicating that the wear scar surface is primarily covered by WC–abrasive particles or a small amount of WC–abrasive debris, generated by friction. The abrasive particles of the local area form third-body tribo-layers under the extrusion action of tribo-pairs. The adhesion of small particles and the appearance of the tribo-layers isolate the direct contact of the tribo-pairs, further protecting the coating from wear.

Because of the repeated chiseling effect of particles on the coating, the coating surface is covered with gouging pits (Figure 5d,g), and the bonding force between the binder phase and the hard phase is reduced, and eventually, the binder phase is peeled off, and the hard phase is pulled out [27], and most of the coating surface is destroyed. It is worth mentioning that there are local tribo-layers in the middle area of the wear scars under a 36 μm particles condition (Figure 5e), which slows down the wear of the subsurface materials to some extent. However, there are still many severe spalling pits left by the hard phase pull-out in the absence of friction layers (Figure 5h). The above tribo-layers contain more Ni and Cr coating elements than the tribo-layers under small particle conditions (Zone D in Figure 5i), indicating that under large particle conditions, the wear debris generated by the material shedding on the coating surface becomes part of the abrasive particles and participates in the wear.

**Figure 6:**
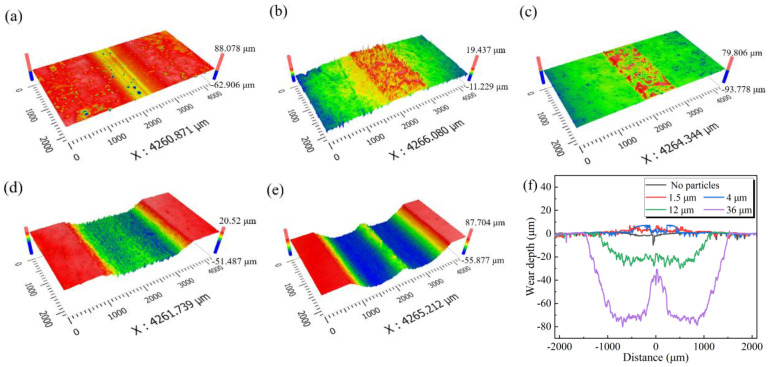
Three-dimensional morphology of the coating wear scars under different particle sizes: (**a**) no wear; (**b**) 1.5 μm; (**c**) 4 μm; (**d**) 12 μm; (**e**) 36 μm; (**f**) two-dimensional contours of each coating wear scars.

Figure 6 shows the three-dimensional topography, and the comparison of the two-dimensional profiles of the coating wear scars under different particle size conditions. Obviously, in the absence of abrasive particles, the wear scar area is relatively flat, with no discernible quality loss (see Figure 6a). When small particles are used, the wear scar has obvious protrusions when compared to the non-wear area (Figure 6b,c). These are the third-body layers formed by the abrasive particles compacting and adhering to the surface. However, in the case of large particles, there are no obvious third-body layers in the wear scar area, which is replaced by deep grooves. Among them, the shape of the wear scar under the condition of 36 μm particles is “W” (Figure 6e,f). This is because the large particle size has a greater probability of entering both sides of the tribo-pairs and participating in the wear. After a certain wear time, particles are discharged from the tribo-interface, forming a steady state of entry–wear–push, thus accelerating the wear on both sides of the tribo–pairs. Bits of particles are pushed to the center of the wear scars, where they form tribo–layers, reducing wear loss and protecting the coating’s middle region (Figure 5e). According to the two–dimensional profiles shown in Figure 6f, as the WC particle size increases, the wear scar depth first increases and then sharply decreases, reaching its maximum (around 77.8 μm) with 36 μm particles.

### 3.4. Surface Morphology of Counter-Balls

**Figure 7:**
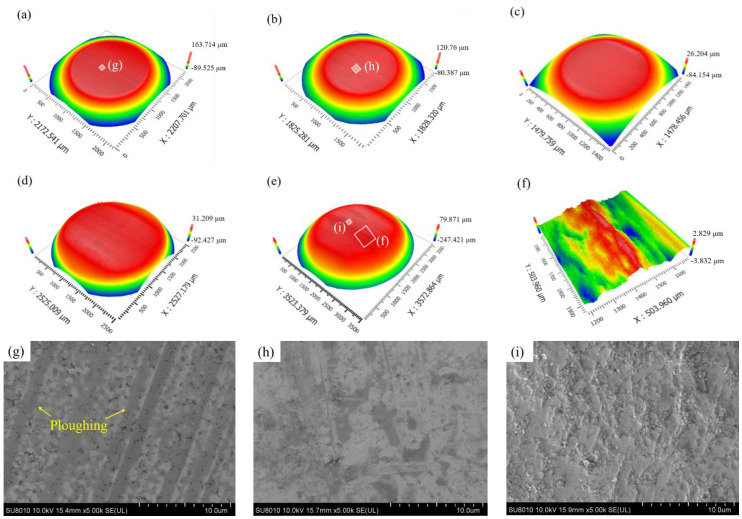
Wear scar morphology of WC ball under different particle size conditions: Three-dimensional morphology (**a**,**g**) no particles; (**b**,**h**) 1.5 μm; (**c**) 4 μm; (**d**) 12 μm; (**e**,**i**) 36 μm; (**f**) the micro-region of (**e**).

Figure 7 presents the wear scar morphologies of counter WC balls under different particle size conditions. Under no-particle and small-particle conditions, the worn surface is relatively flat, with a clear boundary (Figure 7a–c). The tribo-interface is uniformly worn and characterized by ploughing when no particles or only a small amount of abrasive particles are present (Figure 7g). The tribo-layers and adhering particles formed at the interface also isolate the direct contact between tribo-pairs without causing significant stress concentration, which slows the WC ball wear and results in a relatively smooth worn surface (Figure 7h). However, under the condition of large particles, the boundary of the wear area of the WC ball is blurred, and the worn surface then features a significant number of irregular pits of varying depths, showing the phenomenon of “uneven wear” (Figure 7d,e). This is primarily due to the uneven gouging of the contact surface by the majority of the abrasive particles concentrated at the surface edge, resulting in a wear scar with a large number of gouging pits (Figure 7i). The above phenomena are all matched with the wear morphology and characteristics of the counter-coatings.

### 3.5. Wear Loss of Tribo-Pairs

**Figure 8:**
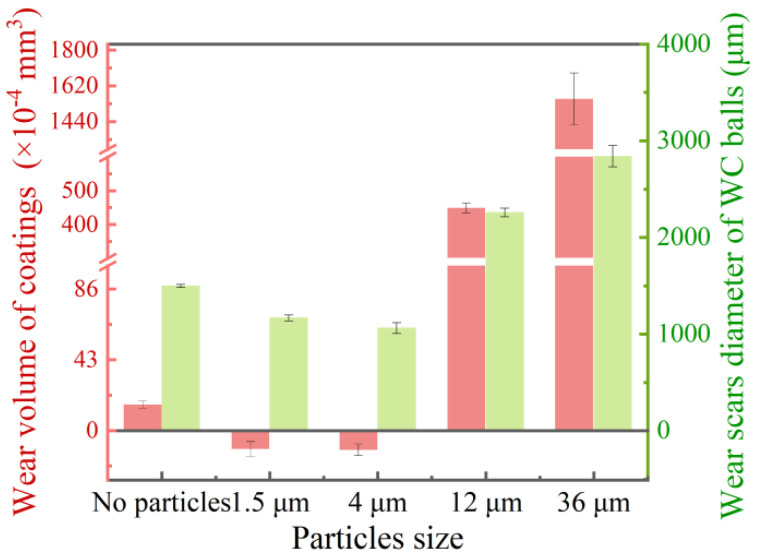
Wear loss of tribo-pairs: Left: wear volume of coatings; Right: wear scars diameter of WC balls.

Figure 8 depicts the wear volume of the coatings and the diameter of the WC balls’ wear area, which is presented in Figure 7a–e. It can be seen that the wear loss of two tribo-pairs has similar variation rules as the particle size increases. Specifically, under the condition of no particles, the material loss is slight. Under the condition of small particles, the wear diameter of the WC balls is lower than that under the condition of no particles, and the coating has a negative wear phenomenon, which is generated by the accumulation and agglomeration of abrasive particles on the coating surface, indicating that the third-body particles play a role in slowing down the wear and protecting the coatings during the friction process. On the contrary, the wear scar diameter of the WC balls and the wear volume of the coating under the condition of large particles are higher than those without particles, and the increase is as high as about 51% and 90%, respectively. It is worth mentioning that the wear volume of the coating under 12 μm and 36 μm particles increased by more than 30 and nearly 100 times compared with the condition without particles, respectively. The above results indicate that large-sized particles can aggravate the wear and destroy the coating during the friction process.

## 4. Discussion

**Figure 9:**
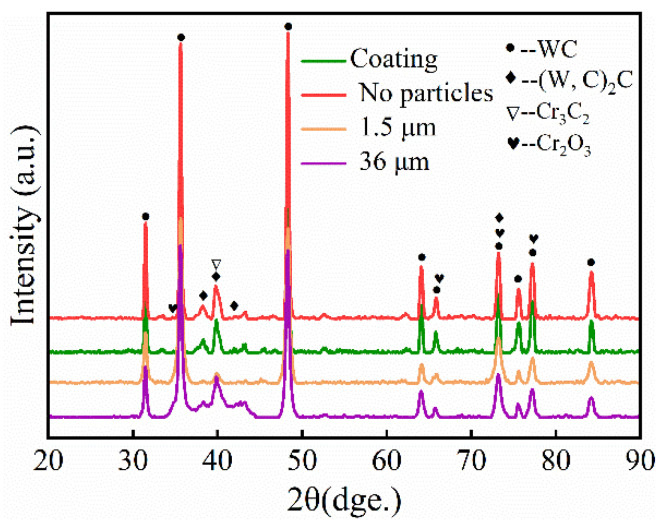
XRD patterns of coating wear scar micro-domains.

Figure 9 displays the XRD patterns of the coating and wear scar micro-domains. The primary phases of the coating are WC, (W,C)_2_C, Cr_3_C_2_ and Cr_2_O_3_, as can be observed. Almost no Ni binder was detected in the coating, and the disappearance of Ni can be attributed to the rapid cooling and the formation of the amorphous Ni phase in the 2θ range of 37°~46° [24,28]. In addition, the (W,Cr)_2_C formed in the coating as the second carbide phase, which is stronger than the Cr_3_C_2_ phase, indicating that the W/Cr ratio undergoes severe stoichiometric changes during spraying. The formation of the (W,Cr)_2_C phase is the result of the pyrometallurgical reactions that may occur even during sintering [15,19]. The high temperature of the spraying process causes the decomposition of carbides in the coating: Cr_3_C_2_ is decarburized and oxidized to form the Cr_2_O_3_ phases. Cr_3_C_2_, (W,Cr)_2_C and WC are all hard phases, and they are evenly distributed in the coatings, which play a role in dispersion strengthening and increase the hardness of the coating [29]. By comparison, it is found that when the particles participate in wear, the WC peak intensity on the coating surface is higher than that of the non-wear and dry-friction coatings. This is ascribed to the detection of surface-adhered WC particles. Under different conditions of particle size, the worn surface of the coatings has similar phase compositions.

**Figure 10:**
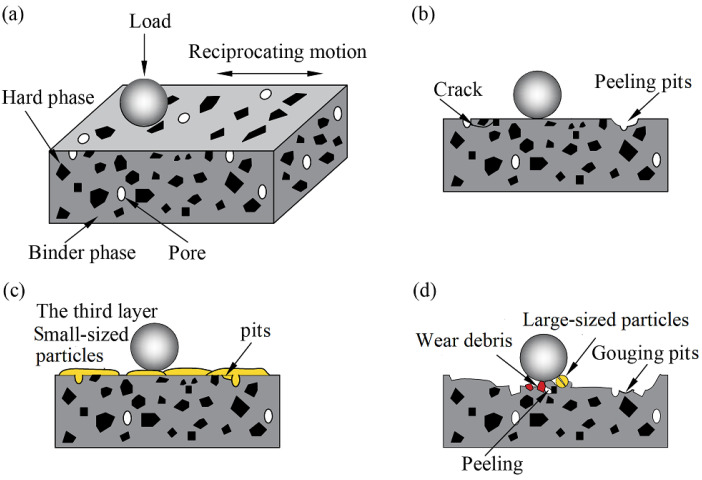
Schematic diagram of wear mechanism: (**a**) initial state; (**b**) no particles; (**c**) small-sized particles; (**d**) large-sized particles.

The COF of tribo-pairs first decreased and then increased with the increase in particle size, and was relatively low under the condition of large particles (Figure 4). However, a low COF does not mean high wear resistance, and there is no direct relationship between the two. Compared with the particle-free condition, it is found that the wear loss of coatings and counter-balls with the participation of small particles is smaller (Figure 8), and the particle environment has a certain protective effect on the tribo-pairs. However, the wear losses of two tribo-pairs under large particle conditions are much larger than under other conditions. The difference in the wear phenomena of the WC–Cr_3_C_2_–Ni coating is determined by the material composition and abrasive particle size. Since the coating is composed of hard phases and binder phases, the hardness of the two phases is quite different. During the reciprocating motion, the WC ball produces alternating shear stress and compressive stress on the coating surface, which repeatedly acts on hard phases and binder phases (Figure 10a). However, different contact states will lead to different wear results [30]. The shear stress on the coating surface and the contact state of tribo-pairs will change due to the size of the particles participating in the friction, thus resulting in different wear mechanisms. The above complete results will be used to investigate the damage behavior and material removal mechanism of the WC–Cr_3_C_2_–Ni coatings under various abrasive particle sizes.

When there are no particles (Figure 10b), the tribo-pair interface is in direct contact, and the hard phases undergo elastic-plastic deformation due to shear stress. The surface of the ball exhibits obvious ploughing characteristics (Figure 7g). Shear stress, on the other hand, acts continuously on the carbide hard phases and binder phases, and fatigue microcracks are prone to forming at the bonding interface of binder phases and hard phases or in the pores [31]. It is worth noting that these cracks mainly propagate along the binder phases and eventually form fatigue spalling. Additionally, these spallings are ground between the tribo-pairs and become hard abrasive particles, resulting in a large number of ploughing on the surface of the WC ball [32]. However, when the crack encounters hard phases, such as WC, Cr_3_C_2_, or W_2_C, its propagation is effectively stopped, and delamination appears (Figure 5a). On the other hand, the binder phases are oxidized during the reciprocating friction process, and the oxide layers shear with the subsurface layers, which aggravates the cracking and delamination of the coating. Therefore, under a no-particles condition, the coating’s wear mechanism is primarily fatigue wear and a slight oxidative wear.

**Figure 11:**
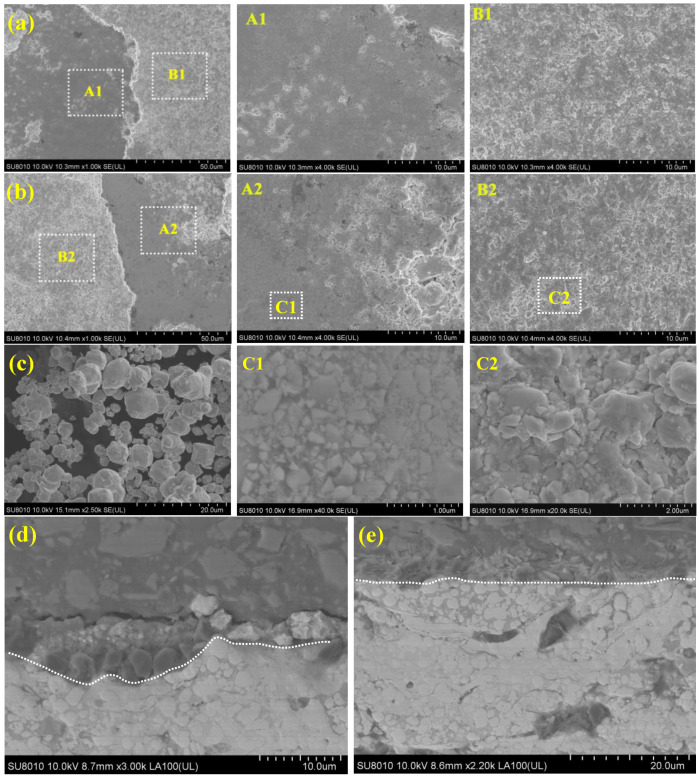
(**a**) Morphology of coating wear scar under 1.5 μm: (**A1**) tribo-layer area, (**B1**) non-tribo-layer area. (**b**) Morphology of coating wear scar under 4 μm: (**A2**) tribo-layer area, (**B2**) non-tribo-layer area. (**c**) Morphology of 4 μm particles; (**C1**) enlarged view of tribo-layer area; (**C2**) enlarged view of non-tribo-layer area. (**d**) The cross-section of coating after rubbing under small-sized particles condition; (**e**) the cross-section of unworn coating.

The morphology of the tribo-layer areas and non-tribo-layer areas in Figure 5b,c and the cross-section of the coating after rubbing under the small-sized particles condition have been presented in Figure 11. The coating surface, after rubbing under the small-sized particles condition, is mainly composed of a smooth tribo-layer (Figure 11a(A1,A2)) and particle dispersion area (Figure 11a(B1),b(B2)). A3 and B3 have partially enlarged views of the smooth tribo-layer and the particle dispersion area, respectively. It can be seen from the comparison that both regions are covered by particles. The morphology of the 4 μm abrasive particles before (Figure 11c) and after wear (Figure 11b(C1,C2)) has been compared, and it was found that the size of the particles on the coating wear scar was significantly reduced. It indicated that the high-contact stress during the test led to the breakage of the small particles. Due to the small particle size being smaller than most of the pores of the coating, some particles were deposited on the coating surface and in the pores, which indirectly affects the wear resistance of the coating [33]. A closer look reveals that the particles composing the smooth tribo-layers are much smaller than those in the particle dispersion zone, and the tribo-layers are likely to be formed by the plasticization of broken small particles under the action of high-contact stress. This tribo-layer is called the “glaze layer”, which slows down the tribo-pairs’wear. To further understand the formation of tribo-layers, a cross-sectional analysis of the wear scar was performed under small particle conditions (Figure 11d). In addition to the wear debris formed by the partially exfoliated coating blocks, it was also found that the spalling pits of the coating wear scars were filled with broken particles, which have similar characteristics to the coating wear scars. The unworn coating has a relatively smooth interface (Figure 11e). Thus, it was confirmed that the particles involved in wear are broken and filled in the spalling pits or attached to the coating surface. In detail, the small abrasive particles entered the tribo-interface and peeled off the coating surface, thus, the COF fluctuates greatly in the early stage of friction. As the wear progresses, the abrasive particles are gradually broken under the high-contact stress action before being pushed out of the interface, and then filled in the spalling pits and covered on the worn surface (Figure 10c), and the COF decreases accordingly. After the abrasive particles are refined to a certain extent, plasticization occurs to form a tribo-layer.

In the large particle conditions (Figure 10d), the particles easily enter the tribo-interface, and the COF is lower than that in other conditions due to the rolling contact of a large amount of abrasives (Figure 4). The abrasive particles are fractured and broken during the reciprocating cycle, and at the same time, they constantly chip away the binder phases, and finally, hard phases are pulled out from the coating. In addition, abrasive particles and the wear debris, composed of shedding of binder and hard phases, mixed together with the tribo-pairs to form a three-body abrasive wear state “WC ball-mixed abrasive-coating”. On the one hand, for the 12 μm abrasive particles, the abrasive rolls relatively smooth over the entire wear surface, resulting in a lower COF. On the other hand, these mixed abrasives also chisel the tribo-pairs, which accelerates the wear of the coatings with a higher wear loss (Figure 8). However, for the slightly larger abrasive particles (36 μm), after the abrasive particles enter the wear interface, a larger shear force is required to push the particles and establish the rolling state. A small amount of damaged abrasive particles, which can move to the middle of the wear scars, have a relatively weak chiseling effect, thus forming a small area of ”glaze layers”, which has a protective effect on the coating. In other areas, fresh surfaces are always exposed due to the continuous chipping of abrasives, and stable glazes cannot be formed (Figure 5e). As a result, uneven wear appears; that is, the wear on both sides of the wear scar is serious and shows a “W”—shaped wear morphology (Figure 6e,f).

## 5. Conclusions

In this work, WC–10Cr_3_C_2_–12Ni coatings were prepared by using the HVOF spraying technology, and the three-body abrasive wear of the coating under different WC particle sizes was studied. There were significant differences in the tribological behavior and material removal mechanism of the coatings under different particle size conditions.

1. The WC–Cr_3_C_2_–Ni coating deposited by the HVOF technology contains phases, such as WC, W2C, Cr_7_C_3_, Cr_3_C_2_, Cr_2_O_3_ and Ni. The coating has a dense and uniform tissue and is closely bonded with the substrate. The average nanohardness of the coating is about 16 GPa, nearly five times higher than that of the 20# steel substrate.
2. The COF of tribo-pairs decreases and then increases as the particle size increases. Under 12 μm and 36 μm particle conditions, the COF is reduced by about 60% and 44% in comparison with dry friction, respectively, and the corresponding increase in the coating wear loss is more than 30 times. Furthermore, the wear loss trends for the particle size for friction balls and the coatings are similar.
3. The particle size has a direct relationship with the coating wear. The surface of the coating appears to wear slightly in the absence of particles, and the wear mechanism is primarily a fatigue wear and minor oxidation wear. Small-sized particles were crushed and adhered to the coating surface, whereafter the extremely fine particles were plasticized to form a large area of tribo-layers by a high stress that reduced the wear and provided protection for the coatings. The protective effect of the abrasive particles fades as the particle size increases, while the chiseling effect intensifies, resulting in a significant tribo-pair loss.

## Figures and Tables

**Table 1:** HVOF parameters.

Oxygen Gas, 0.65 MPa	Propane Gas, 0.4 Mpa	N_2_ Feed Gas, 0.6 Mpa	Torch Distance
10 m^3^/h	1.5 m^3^/h	1.0 m^3^/h	170 mm

**Table 2:** The parameters of friction and wear test.

Load	Sliding Distance	Reciprocating Frequency	Cycles	WC Abrasive Size
50 N	5 mm	4 Hz	25,000 N	1.5, 4, 12, 36 μm

## Data Availability

The data presented in this study are available on request from the corresponding author.
